# Viability and Resilience of Languages in Competition

**DOI:** 10.1371/journal.pone.0008681

**Published:** 2010-01-26

**Authors:** Laetitia Chapel, Xavier Castelló, Claire Bernard, Guillaume Deffuant, Víctor M. Eguíluz, Sophie Martin, Maxi San Miguel

**Affiliations:** 1 Laboratoire d'Ingénierie pour les Systèmes Complexes, Cemagref, Aubière, France; 2 IFISC (CSIC-UIB) Instituto de Física Interdiciplinar y Sistemas Complejos, Palma de Mallorca, Spain; Aarhus University, Denmark

## Abstract

We study the viability and resilience of languages, using a simple dynamical model of two languages in competition. Assuming that public action can modify the prestige of a language in order to avoid language extinction, we analyze two cases: (i) the prestige can only take two values, (ii) it can take any value but its change at each time step is bounded. In both cases, we determine the viability kernel, that is, the set of states for which there exists an action policy maintaining the coexistence of the two languages, and we define such policies. We also study the resilience of the languages and identify configurations from where the system can return to the viability kernel (finite resilience), or where one of the languages is lead to disappear (zero resilience). Within our current framework, the maintenance of a bilingual society is shown to be possible by introducing the prestige of a language as a control variable.

## Introduction

The study of language dynamics using computer simulations has become a research field of increasing interest in the scientific community. Models studying language dynamics range from social impact theory applied to language competition [1] to genetic approaches for the evolution of universal grammar [2]. We are here interested in the problem of language competition, i.e., the dynamics of language use among a population of interacting agents speaking different languages. Around 50% of the 6000 languages spoken today are in danger and will disappear during the current century according to the recent studies in language contact [3]. Beyond Weinreich's *Languages in contact* [4], several studies in sociolinguistics have addressed questions regarding the level of endangerment of specific languages [5] and the challenge to find a common pattern that might relate language choice to ethnicity, community identity or the like [6]. Lately, the need to provide a quantitative analysis in the field of sociolinguistics is getting an increasing attention [7]. This fact has triggered an effort in order to model and understand the mechanisms within scenarios of language competition: some models study the competition between many languages in order to reproduce the distribution of language sizes in the world in terms of the number of speakers [8], [9]; while others focus on the case of language contact between few languages (for a review see Refs [10], [11]). In particular, Abrams and Strogatz [12] proposed a simple mathematical model of competition between two languages. The model describes the system by aggregated variables that represent the fraction of speakers of each language, where a higher local density of speakers and a higher prestige, the relative status of a language, tend to increase the density of speakers of a language. The analytical study of the model and the fitting to real data from the competition between Quechua-Spanish, Scottish Gaelic-English and Welsh-English, predict that the coexistence of two languages is unstable, irrespective of the prestige of the languages and their initial density of speakers in the model, in contrast to the evidence that bilingual societies exist today. The paper finished with the following remarks:

Contrary to the model's stark prediction, bilingual societies do, in fact, exist. […] The example of Quebec French demonstrates that language decline can be slowed by strategies such as policy-making, education and advertising, in essence increasing an endangered language's status. An extension to [the model] that incorporates such control on 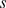 through active feedback does indeed show stabilization of a bilingual fixed point.

Several modifications and extensions of this model of language competition have investigated deeper this problem: (i) developing agent-based models in order to study the behavior of the model in regular networks [13], in which the path to a final scenario of extinction of one of the languages is analyzed in finite size systems; (ii) introducing geographical dependencies in terms of a reaction-diffusion equation, which allow the survival of the two languages, with speakers of different languages mostly located in different geographical areas [14], [15]; (iii) implementing Lotka-Volterra type modifications to the original model which can lead to a scenario of coexistence of the two languages in the same geographical area [16]; (iv) introducing bilingualism in the model: individuals can use both languages [17], [18]. In this last extension [18], and in the same parameter setting studied by Abrams and Strogatz, introducing bilingualism keeps the coexistence of both languages unstable. This extension of the model has been extensively studied and compared to the seminal model of Abrams and Strogatz for the case of socially equivalent languages and linear dependence on the density of speakers [19]. The analysis has been done in agent based models in finite systems where social structure has been taken into account using complex social networks. The models have been studied in two-dimensional regular lattices and small-world networks [19], as well as in networks with community structure [20], [21].

The prestige of a language has been considered as one of the main factors affecting language competition since Labov's *Sociolinguistic Patterns* [22]. It measures the status associated to a language due to individual and social advantages related to the use of that language, being higher according to its presence in education, religion, administration and the media. Minett and Wang [23] defined simple strategies for modifying the prestige to maintain the coexistence of the two languages, following the remarks of the seminal work quoted above [12]. Beyond this initial effort in proposing simple strategies to foster language coexistence, the aim of this work is to provide a more general approach to determine the actions on the prestige to maintain the coexistence of both languages.

We adopt a viability theory perspective: viability theory [24] provides theoretical concepts and practical tools, in order to maintain a dynamical system inside a given set of a priori desired states, called the *viability constraint set*. This set represents the “good health” of a system beyond which its safe existence would be jeopardized; in the context of language maintenance, it characterizes the safe coexistence of both languages. The goal of viability theory is to determine policies (viable policies) that always keep the system inside the viability constraint set, rather than to optimize some criterion. The main concept is the *viability kernel*: the set of states, given some possible control actions on the system, for which the system can be maintained inside the viability constraint set. It provides the actual constraints of the system: inside the viability kernel, there is at least one control policy which maintains the system indefinitely inside the constraint set; outside the viability kernel, the system will break the constraint set, irrespective of the policy applied. Moreover, viability theory provides a particularly appropriate framework to define rigorously the concept of *resilience* [25], the capacity of a system to undergo some exogenous disturbances and to maintain some of its dynamical properties. Resilience is often defined within the dynamic systems theory: it can be measured as a function of the time needed to return to equilibrium after a perturbation [26], or as a function of the distance to bifurcation points [27], where these are defined as points where the stability of a fixed point changes. In the viability framework, the desired properties can be defined by viability constraints, and resilience, which refers to viable states, becomes the capacity to drive the system inside its viability kernel when a perturbation pulls it off. It focuses on the ways by which the system can recover from such a perturbation by providing control policies (if any) that will drive back the system to a safe coexistence scenario with a minimal cost of restoration. Applying viability theory to the Abrams-Strogatz model, We identify the configurations for which an indefinite coexistence can be insured, and provide the corresponding action policies on the prestige. Following Ref [25]'s approach, we study the resilience of the model by identifying configurations from where the system can return to a state of coexistence (finite resilience) and other configurations from where one of the languages faces extinction irrespective of the policy applied (zero resilience).

This paper is divided as follows: in the material and methods section, we introduce the Abrams-Strogatz model, first briefly describing the model and making a stability analysis depending on the parameters. We then state the viability and resilience problems. In the results section, we study the viability of the languages by defining action policies that maintain the system within its viability kernel. In the language resilience subsection, we compute the resilience of the two languages using two dfferent criteria. We finally discuss the results and draw some conclusions.

## Materials and Methods

### Language Dynamics: the Abrams-Strogatz Model

**Figure 1:**
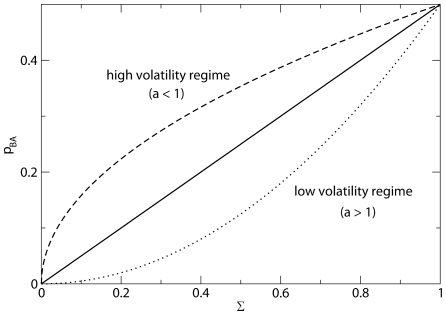
Dependence on the volatility parameter 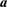 for the transition probability to change from state 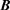 to state 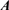, 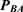. Case of socially equivalent languages (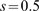). Marginal volatility (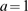, solid line), high volatility regime (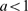, dashed line), and low volatility regime (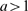, dotted line).

To study the competition between languages in a given population, Abrams and Strogatz proposed a simple model to represent a population with two languages (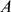 and 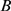) in competition for speakers. Let 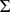 be the fraction of 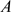-speakers and 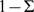 the fraction of 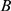-speakers. A 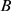-speaker can become an 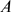-speaker with the probability 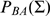, and the inverse event happens with the probability 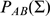. In this way, the time evolution for 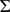 is:
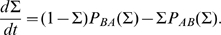
(1)Speakers change their language according to the attractiveness of the other language, which depends on the fraction of speakers and on two parameters: the prestige of the language, 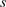, and the volatility, 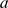. The probability for 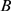-speakers to become 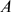-speakers reads:
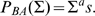
(2)The prestige of language 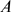 is modelled as a scalar, 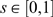 (the prestige of language 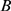 is 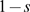), which aggregates the multiple factors affecting the prestige of a language. Notice that the case 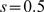 corresponds to the case of socially equivalent languages. The functional form of 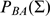 is shaped by the parameter 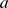, which we define as volatility (see Figure 1). For the case 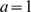, we have the special case of linear transition probabilities (marginal volatility); a high volatility regime is obtained for 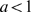, where the transition probabilities are larger than linear (agents are likely to change language); while a low volatility regime is obtained for 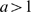 where happens the opposite (agents more rarely change their language). Similarly, the probability for 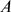-speakers to become 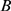-speakers is:
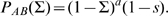
(3)Equations 2 and 3 incorporate the assumption that if a language has no speakers or has zero prestige, the probability for a speaker to change for this extinct language is zero.

Introducing Eqns 2 and 3 in Eqn 1, the Abrams-Strogatz model results in the following population dynamics
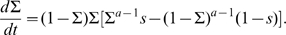
(4)


We focus now on a brief stability analysis of the model. When 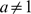, the stability analysis shows that there are three fixed points: 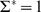 and 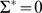 which correspond to consensus in the state 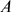 or 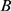, respectively; and the other one corresponds to coexistence:
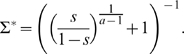
(5)

- For 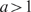, the two first fixed points are stable, and the third one is unstable, leading to a scenario of dominance of one of the languages and extinction of the other.
- For 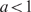 instead, the stability of the fixed points changes: consensus becomes unstable giving rise to the coexistence of the two languages. A change in the status does not change the stability of the fixed points, but changes its value; the higher the difference in the relative prestige, the higher the difference in densities between the two languages in the third fixed point. Notice that the case 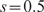 corresponds to the case of socially equivalent languages, and for this case, the transition probabilities (Eqns 2 and 3) become symmetric and the third solution is 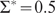 independently of 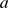.
- For 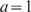, and 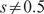, Eqn 4 becomes the logistic-Verhulst equation [13]:
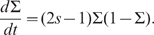
(6)In this case, there exist just two fixed points: (i) 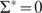 and (ii) 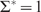. For 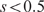, (i) is stable and (ii) unstable while for 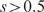 it happens the opposite. For the case 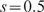, we obtain 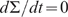 with a degeneracy of fixed points: any initial condition is a fixed point of the dynamics. This special case of socially equivalent languages and linear transition probabilities corresponds to the voter model dynamics, extensively studied in complex networks [28]–[31].

### Language Viability

In this work, we are interested in how active policies in favor of an endangered language might lead to a coexistence of the two languages in competition. Abrams and Strogatz already suggested that [12]:

An extension to Eqn 4 that incorporates such control on 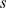 through active feedback does indeed show stabilization of a bilingual fixed point.

We will give evidence of this remark by studying the Abrams-Strogatz model in a viability theory framework.

Viability theory [24] focuses on how to maintain a dynamical system inside a viability constraint set. The system is composed by state variables, that describe the system, and by control variables that allow one to act on it. The *viability constraint set* defines a state set outside which the system escapes from an a priori desired setting. A state is called viable if there exists at least one control function that maintains indefinitely the system inside the viability constraint set; the set of all these viable states is called the *viability kernel*. The viability problem is thus to define a control function that keeps the system viable. On the contrary, for states located outside the viability kernel, all possible evolutions break the constraints in finite time. As shown below, the viability kernel is essential in order to define action policies that maintain viability and the main task in order to solve a viability problem is thus to determine its viability kernel.

When defining the viability constraint set in the case of language competition, in general, in order to characterize a language as endangered, the fraction of people speaking it is not enough: other crucial aspects include the point at which children no longer learn the language as their mother tongue; as well as the increase of the average age of speakers (in an endangered language, eventually only older generations speak the language) [32]. However, these factors are out of the scope of the current approach, and we will assume in this work, as a first approximation, that a fraction of speakers below a critical value becomes an endangered situation. Building up from this point, in the Abrams-Strogatz model, we want to determine all the couples of density of speakers and language prestige which let the coexistence of the two languages. The viability constraint set is defined by setting minimal and maximal thresholds on the density of speakers. Below the minimal threshold, 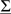, or above the maximal threshold, 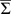, we consider that language 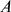, or 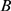 respectively, is endagered, meaning that the system is not viable. We set 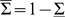 such that there is no need to consider explicitly language 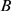: if 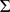 is outside the constraint set, so does 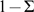.

As it is advocated in Ref [12], we introduce prestige 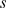 as the control variable. The enhancement of the prestige of an endangered language can be triggered by political actions such as the increase of the prestige, wealth and legitimate power of its speakers within the dominant community, the strong presence of the language in the educational system, the possibility that the speakers can write their language down, and the use of electronic technology by its speakers [3]. The computation of the viability kernel for the Abrams-Strogatz model will allow us to answer questions like: for a given density of speakers, are there action policies performed in favor of the endangered language that will keep the coexistence of the two languages? If the answer is yes, which are convenient policies? To answer this question, Minett and Wang [23] proposed strategies in a simple framework (only two control values are considered). The main advantage of using viability theory is that it provides general tools and methods to determine the set of initial density of speakers for which it is possible to control the system such that the coexistence is ensured.

### Language Resilience

We study the viability of the language model, supposing that one language is endangered when its density of speakers goes below a critical value. However, being endangered does not necessarily mean that the language will disappear. In the resilience problem, we are interested in how to maintain or restore coexistence of the two languages when the system is in danger, meaning that a disturbance pulls it outside the viability constraint set.

As we pointed out in the introduction, resilience is the capacity of a system to restore its properties of interest, lost after disturbances. In this subsection, we define resilience of system Eqns 9 and 10 by considering its capacity to return into its viability kernel when a perturbation pulls it out from it, following Ref [25] definition of resilience.

We are interested in situations of crisis, which take place when the system leaves the viability constraint set. We distinguish two types of states located outside the viability kernel:

- States for which there exists at least one evolution driving back the system to the viability kernel after leaving the constraint set, are called resilient. The system is resilient to a perturbation which leads it into a resilient state;
- States for which irrespective of the control policy applied, the system remains outside the viability kernel, are called non-resilient. The system is not resilient to perturbations leading the system into a non-resilient state.

For states located inside the viability kernel, the resilience is infinite. Reference [25] also introduces the notion of cost of restoration in its resilience definition. This cost measures the distance between the evolution of the state of the system and the property of interest (i.e. being inside the viability kernel). Its definition must fulfill three conditions. First, the cost of an action which keeps the property of interest indefinitely is zero: maintaining this property may lead to some action update, but they are not taken into account in the cost computation. Second, when the property of interest can not be restored, the cost of restoration is infinite. Third, when the property can be restored, the cost is finite. It is often defined by the minimum time the system is outside the viability kernel or the minimal deficit accumulated along the trajectory. Then, the resilience is the inverse of the restoration cost of the properties of interest lost after disturbances. The trajectory starting from 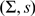 with a minimal cost defines the sequence of “best” action policies to perform, and thus defines the resilience value. Resilience values can be approximated numerically using Ref [33]'s algorithm, which is based on the Ref [34]'s viability kernel approximation algorithm. In the context of language competition, the use of viability theory provides a measure of the cost associated to a policy action which will favor an endangered language.

## Results and Discussion

### Language Viability

We consider three values of the volatility parameter: 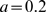, 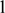 and 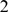. Note that in the case 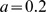 (in general for 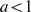), the fixed point corresponding to coexistence of the two languages is stable, and thus no control parameter on s needs to be included to stabilize a bilingual fixed point. However, depending on the difference in the prestige of the two languages, the fixed point might lay outside the constraint set.

#### First case: two prestige values

Following the idea of Minett and Wang [23], we consider first a setting where the control 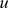 is the prestige 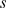 of language 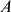, and we restrict the possible values of the control to only two discrete values 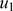 and 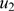. We consider the following viability problem: Find the action policies (a function defining the action in time), such that the dynamical system
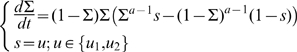
(7)remains in the viability constraint set 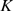:
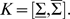
(8)Our aim is to find the set of values of 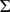 for which there exists at least one control function that keeps the states of the system defined by Eqn 7 always inside the viability constraint set (Eqn 8). The set of all the values of 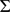 satisfying Eqns 7 and 8 constitutes the viability kernel associated to the model with such control settings, and is denoted 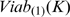.

We will assume that the critical threshold of the density of speakers is 20% of the size of the whole population. Thus we set 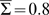 and 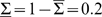, the viability constraint being 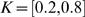. We also suppose that some action can switch the prestige of language 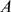 at any time from 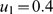 to 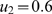. The theoretical boundaries of the viability kernel can be determined analytically. Table 1 gives the boundaries of viability kernels for three values of the volatility 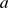: 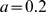, 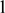 and 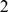. The details and proofs are given in Appendix S1.

**Table 1:** Boundaries of the viability kernel for the dynamics associated to system Eqn 7 and Eqn 8.

	Lower Bound	Upper Bound
**a = 0.2**	0.2	0.8
**a = 1**	0.2	0.8
**a = 2**	0.4	0.6

For 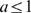, the viability kernel is the whole constraint set. This means that it is possible to maintain language coexistence between 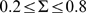, irrespective of the initial density of speakers 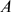 and the initial value of the prestige (given that the initial state belongs to the constraint set, 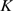). For 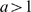, the maintenance is only possible for initial densities of speakers 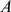 between 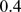 and 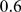. When a state 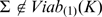, the system will leave the viability constraint set, irrespective of the actions applied.

We are interested now in how frequently policy actions must be performed. We use the heavy control principle, which specifies to change the control only when viability is at stake. The principle of the heavy control algorithm is as follows:

- consider an initial state 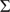 located inside the viability kernel and an initial control 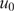;
- anticipate the state of the system at the next time step, keeping the same control;
- if the obtained state is inside the viability kernel, then the control does not change;
- on the contrary, if it is outside the viability kernel, then change the control.

**Figure 2:**
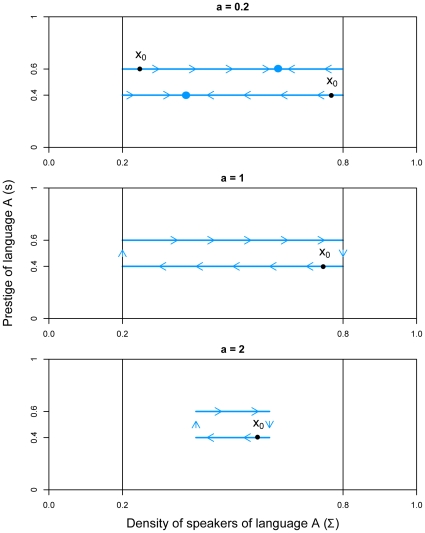
Viability kernels and trajectories that maintain the system viable for 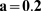, 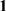 and 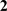. The viability kernels are represented in blue and stable attractors (if any) by dots. Arrows represent the field direction and the controls to choose. For 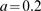, any control is convenient because they lead the system to a stable fixed point. For 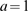 and 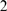, when trajectory lead to a point located outside the viability kernel, the control value must be changed in order to ensure coexistence.

Viability theory guarantees that this procedure maintains language coexistence. However, there may be many action policies that ensure coexistence: the only requirement is that the chosen controls never lead outside the viability kernel. Figure 2 displays viability kernels and control policies. For 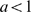, there exists a stable fixed point and the trajectory leads to equilibrium. Starting from any initial density of 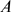-speakers and prestige, there is no need to apply any control policy; the equilibrium is naturally reached. For 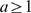, there are no stable fixed points inside the viability constraint set. The control procedure is then applied at each time step: the control is changed only when it leads to a point located outside the viability kernel.

#### Second case: prestige chosen in a continuous interval

In this paragraph, instead of taking only two values, we suppose that the prestige can take any value 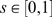 but the action on the prestige is not immediate: the time variation of the prestige 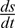 is bounded by a constant denoted 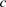. This bound reflects that changes in prestige take time: to reach a prestige value 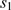 starting from an initial prestige 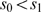, the stakeholder will have to anticipate at least 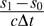 time steps, where 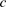 is the maximum change per unit time 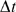. We consider the viability problem to define a function 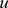 of time, which maintains the dynamical system:
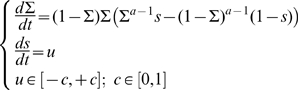
(9)inside the viability constraint set 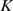:
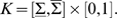
(10)The first step is to determine the viability kernel 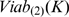, defined by all couples 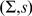 that are solution of the system, Eqn 9, for which there exists at least one control function keeping the system indefinitely inside the viability constraint set defined by Eqn 10.

**Figure 3:**
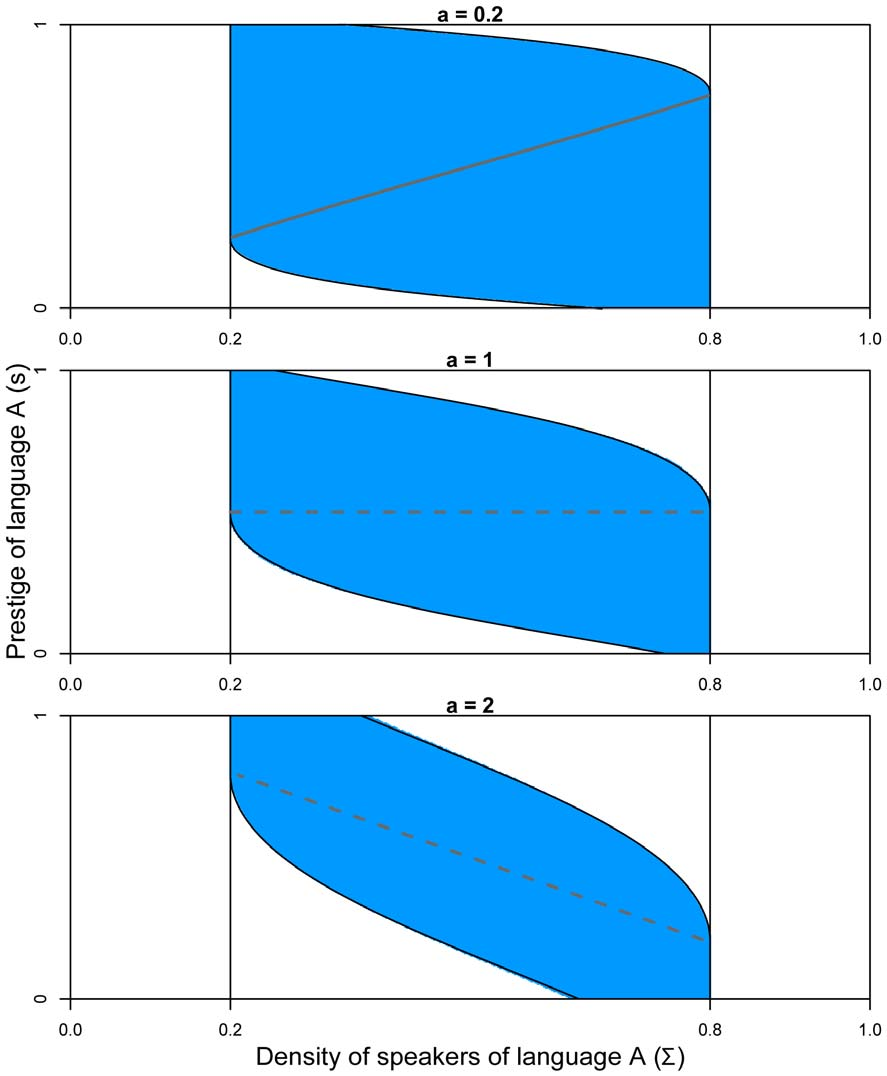
Viability kernel for the Abrams-Strogatz model, with 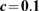 and 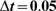. The continuous black lines represent the theoretical curves of the viability kernel, and the area in blue the approximation. The continuous grey line represents stable fixed points and the dotted grey lines unstable fixed points.

We still assume again that the critical threshold of the density of speakers is 20% of the size of the whole population. Therefore, the viability constraint set is 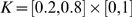. The theoretical boundaries of the viability kernel can be computed analytically (Appendix S2). In general, there exists no explicit formula to define the viability kernel boundaries and algorithms have been proposed to approximate them. In this paper and in addition to the theoretical boundaries, we approximate the viability kernel using the algorithm described in Ref [34], that considers the dynamics in discrete time 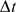. The obtained approximation enables us to use a simpler heavy control procedure. Figure 3 shows the analytical and approximated viability kernels of the system for 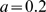, 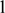, and 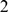. The thick grey lines corresponding to the fixed points of the dynamics has been obtained using Eqn 5. We set 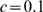, which means that the time variation of the prestige cannot be higher than 10%. The figure shows how for states with a low 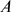 or 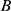-speakers density, the prestige associated to this language must be strong enough to maintain viability. In situations where the density of one language is high, smaller values of its associated prestige also give raise to viable situations. On the contrary, non-viable states correspond to situations where the density of one language and its associated prestige are low at the same time. In this case, if the actions in favor of this language come too late, its density of speakers will get below the critical threshold 20% while the other will spread through the majority of the population (above 80%). As 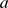 increases, the viability kernel shrinks. Indeed, the higher the parameter 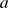, the more rarely agents change their language (low volatility regime). The impact of the change on the prestige is then lower as 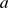 increases, which means that when a language is close to the boundary of the viability kernel, even with the maximal government action, the effect on the density of speakers will be too slow to avoid leaving the viability constraint set. On the contrary, as 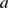 decreases, agents are likely to change their language (high volatility regime) and to restore coexistence. Note that for 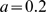, the viability kernel is not the whole constraint set: non-viable states reach a stable fixed point located outside 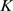.

The control procedure models an action to enhance the prestige of an endangered language, and we assume that such an action is costly. Therefore, if among different possible action policies to maintain language coexistence, doing nothing keeps the system in a viable situation, we assume that this strategy will be chosen in order to reduce costs. In other words, we suppose that, if several situations with 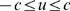 lead to viable situations, the best choice is 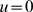. The principle of the control algorithm is roughly as follows:

- consider an initial state 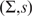 located inside the viability kernel;
- anticipate the trajectory in the next time steps, by considering 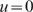;
- if the obtained state is located inside the viability kernel, do not change the control;
- otherwise, choose a control that brings the system away from the viability kernel's boundary as much as possible.

**Figure 4:**
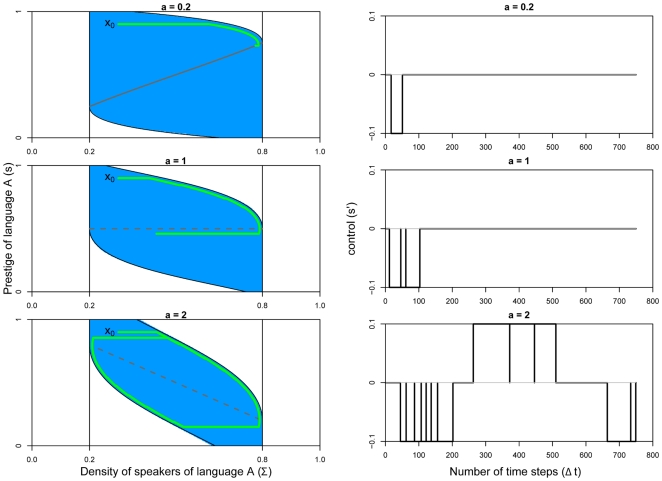
(Left panel) Examples of trajectories (in green) starting from an initial state 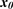 for three values of 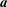 (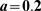, 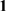 and 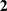), and (right panel) evolution of the control, with 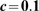. The continuous grey line represents stable fixed points and the dotted grey line unstable fixed points.

This control procedure is described in more details in Ref [34]. We use here the viability kernel approximation boundary instead of the analytical one because it makes easier to check if the anticipation of the trajectory leads to a point outside the kernel and to approximate the distance to the viability kernel boundary. Figure 4 presents some examples of trajectories for three different values of 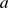, and the time evolution of the control (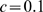), during 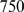 time steps. For 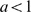, there exist stable fixed points corresponding to coexistence of the two languages and the dynamics settles there, keeping 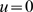 along the trajectory. For 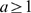 instead, there are no stable fixed points inside the viability kernel, and the control procedure must be applied at each time step. As long as the trajectory is far away from the kernel's boundary, the control is kept to zero; when it approaches the boundary, the control that brings the system away from the boundary corresponds to the maximum value of the control with the appropriate sign, 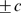.

### Language Resilience

In this subsection, we deal only with the second case, where the prestige is chosen on a continuous interval.

#### Determining the resilient and non-resilient states

All the states can undergo a disturbance. For instance, immigration: people speaking language 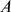 exile to another country, hence the density of 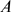-speakers reduces dramatically in the home country, and increases in the destination country. Another perturbation to the system can be due to an abrupt change in the prestige of a language because of political actions such as invasion, occupation, etc. The states resulting from disturbances might bring the system outside the constraint set, leading to situations where the density of speakers is lower than the minimal threshold or higher than the maximal threshold. Thus, we consider now the set of all the possible situations 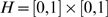, where the first dimension represent the density of speakers of language 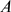 and the second the prestige of language 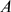, and we study the resilience of the system in 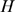.

**Figure 5:**
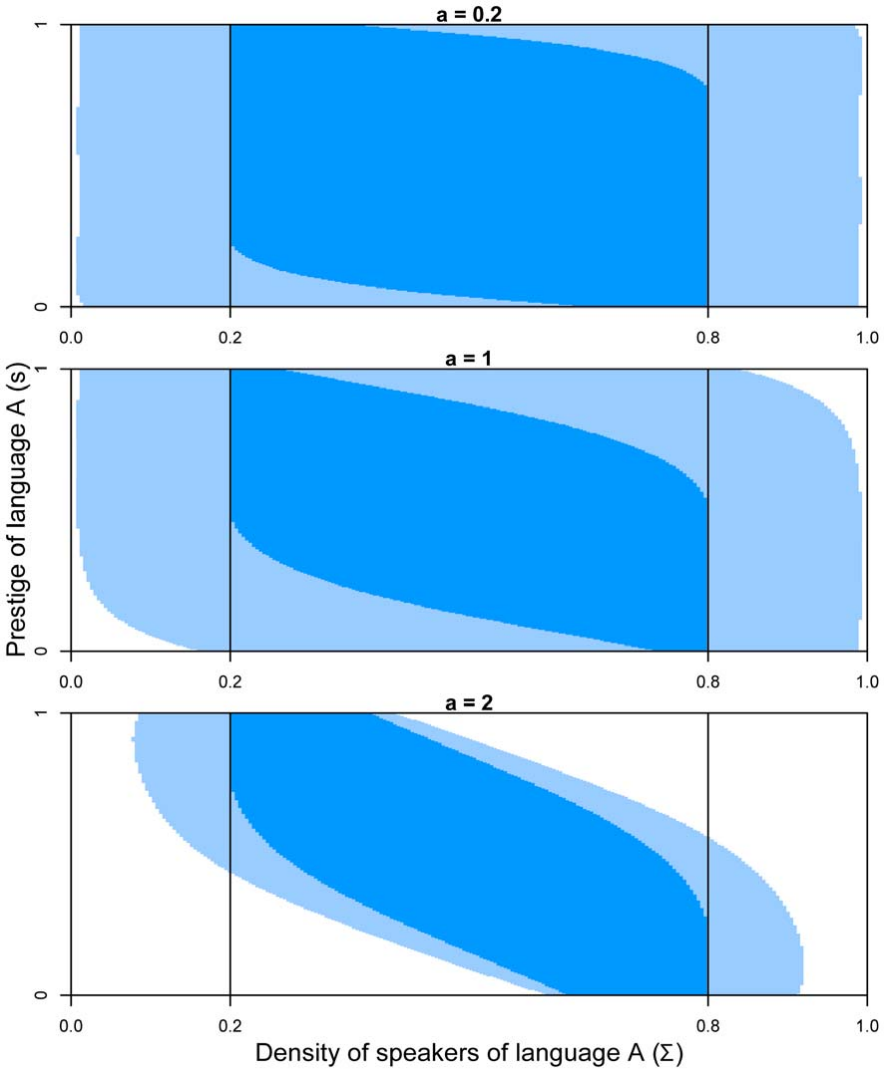
Resilient (blue) and non resilient states (in white) in the model associated to dynamics Eqn 9 with constraint set Eqn 10, for three values of 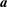: 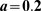, 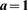, 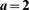. Viability kernel is in dark blue.

First, we determine the set of states of infinite resilience, that are the states located inside the viability kernel of the system defined by Eqn 9 associated to constraint set defined by Eqn 10. It corresponds to the dark blue area on Figure 5. Then, we look for all the states for which at least one evolution drives the system back to the viability kernel after spending a finite time in the critical area 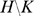 (where 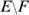 is the complementary set of the set 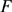 in the set 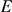). These are the resilient states, in colored light blue in Figure 5. Note that states located in 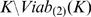 can have a finite resilience: when coming back towards 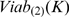, the trajectory leaves the constraint set and reaches 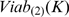 after spending time in the critical area. The states that, irrespective of the applied policy, remain outside the viability kernel are in the white zone. For these states, the desired level of language coexistence is impossible and resilience is zero (given the assumed value of 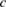, which limits the effect of action).

In Figure 5, we show the resilient and non-resilient states for 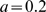, 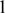, and 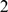. For a small value of 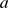, all the states are resilient, except 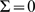 and 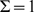, irrespective of the value of 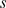. As we pointed out previously, the fixed point corresponding to coexistence is stable for 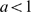. Therefore, the desired level of coexistence for the two languages is ensured or can be reached, irrespective of their initial density of speakers and their prestige, except when a perturbation leads to a situation where one language is already extinct. For 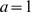, nearly for all the initial density of speakers and prestige, reaching the desired level of languages coexistence is possible, except if the initial state represents a large density of speakers of language 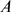 associated with high prestige (language 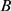 becomes extinct, irrespective of the action applied) or vice versa. For 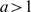, the set of resilient states becomes smaller as it can be seen in Figure 5. The larger the value of 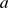, the smaller the set of resilient states is. Indeed, as mentioned before for the shrinking of the viability kernel, a high value of a means that agents rarely change their language and the effects of increasing or decreasing the prestige of a language become less effective.

#### Computing resilience values

As we pointed out previously, the resilience value is then defined as the inverse of its restoration cost. There exist several ways of defining a cost of restoration, depending on the situations and the point of view. We studied two possibilities for the cost: on the one hand, we considered that the time needed to restore viability is the only ingredient under consideration, the cost value is then the time the system is outside the viability kernel. The cost function 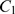 that associates to a state 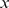 the minimal cost of restoration among all the trajectories starting from 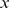 is defined by:
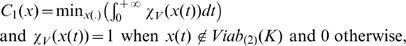
(11)where 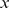 represents the state 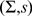, 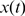 is the state at time 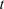 and 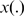 is the trajectory starting from this state. Hence the cost value is zero when the system is inside the viability kernel. On the other hand, we considered a more complete cost function composed of two terms: the first one that accounts for the time the system is not viable, and the second one, representing the distance to the viability constraint set. This cost function, denoted 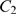, thus associates the time of restoration and the measure of the density of speakers above or below the thresholds of the viability constraint set:
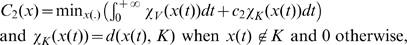
(12)where 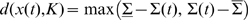 measures the distance between the density 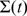 at time 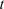 and the density thresholds. Equation 12 takes into account that the cost of restoration of a state near extinction is more costly than the one for states located near the boundary of 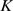. Parameter 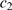 reflects the relative weight of each cost, fixing the cost of being far from 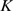 relatively to the time spent outside the viability kernel.

**Figure 6:**
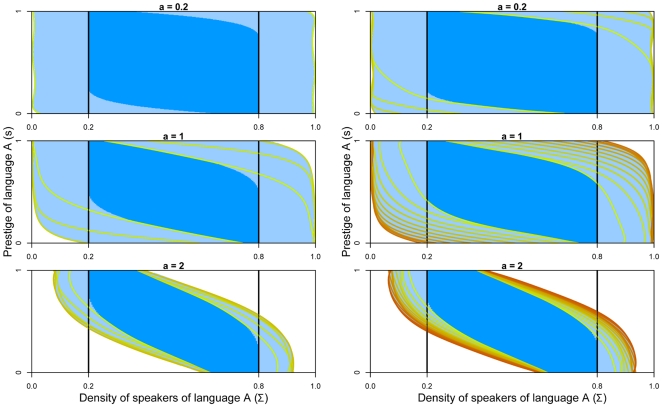
Resilience values of the Abrams-Strogatz model. In dark blue, the viability kernel; between the level lines (light blue area), the cost of restoration is finite (one level line corresponds to a cost of 4.8 and the darker the line, the higher the cost); in the white area, the cost is infinite and the resilience is zero. (Left panel) Cost function 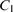 (Eqn 11); (Right panel) cost function 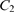 (Eqn 12).

Figure 6 compares resilience values for the Abrams-Strogatz model for different values of 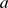, and for the two cost functions defined (with an arbitrary cost parameter 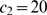 for the second cost function). The difference of cost between two iso-cost curves is 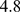, and therefore the difference in resilience is 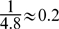 (the 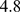 value is arbitrary and is linked to the parametrization of the algorithm in Ref [33]). The darker the line, the higher the cost value is. In the white area, cost is infinite, meaning that restoring coexistence of both languages is impossible. For 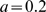, the maximal cost of restoration is equal to 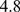 for cost function 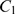 defined by Eqn 11 and 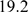 for the cost 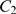 defined by Eqn 12. The cost associated to the function defined by Eqn 12 is bigger than the one associated with Eqn 11 because it introduces an additional part (the distance to viability) on the final cost. For 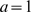, the maximal cost of restoration is more important (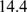 for Eqn 11 and 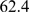 for Eqn 12). For 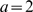, the resilient zone is smaller and the costs of restoration are larger (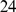 for Eqn 11 and 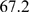 for Eqn 12). This means that for higher values of 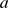, where the resilient set is smaller, the cost of restoration is larger: there are less resilient situations and the action policies to perform in order to restore viability are the most costly.

#### Determining action policies to restore viability at minimum cost

Computing resilience values is instrumental to define action policies that drive back the system inside the viability kernel. Here, we use an optimal controller instead of a heavy controller: we do not look for one action policy that keeps the system in a resilient state, but we define a sequence of actions that allows the system to return to the viability kernel at the lowest cost of restoration. It can be shown (see Ref [33]) that choosing the action that decreases the cost at each step (or increases the resilience), minimizes the whole cost of restoration. Hence, theoretically this approach also provides a means to compute resilient policies, which minimizes the cost of restoration along the trajectory. The procedure is roughly as follows:

- consider an initial state 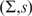 for which resilience is finite;
- choose the action policy that decreases the cost at maximum at each time step, until the trajectory reaches the viability kernel;
- once the state is viable, use the heavy control procedure described previously to ensure the indefinite maintenance of the system.

**Figure 7:**
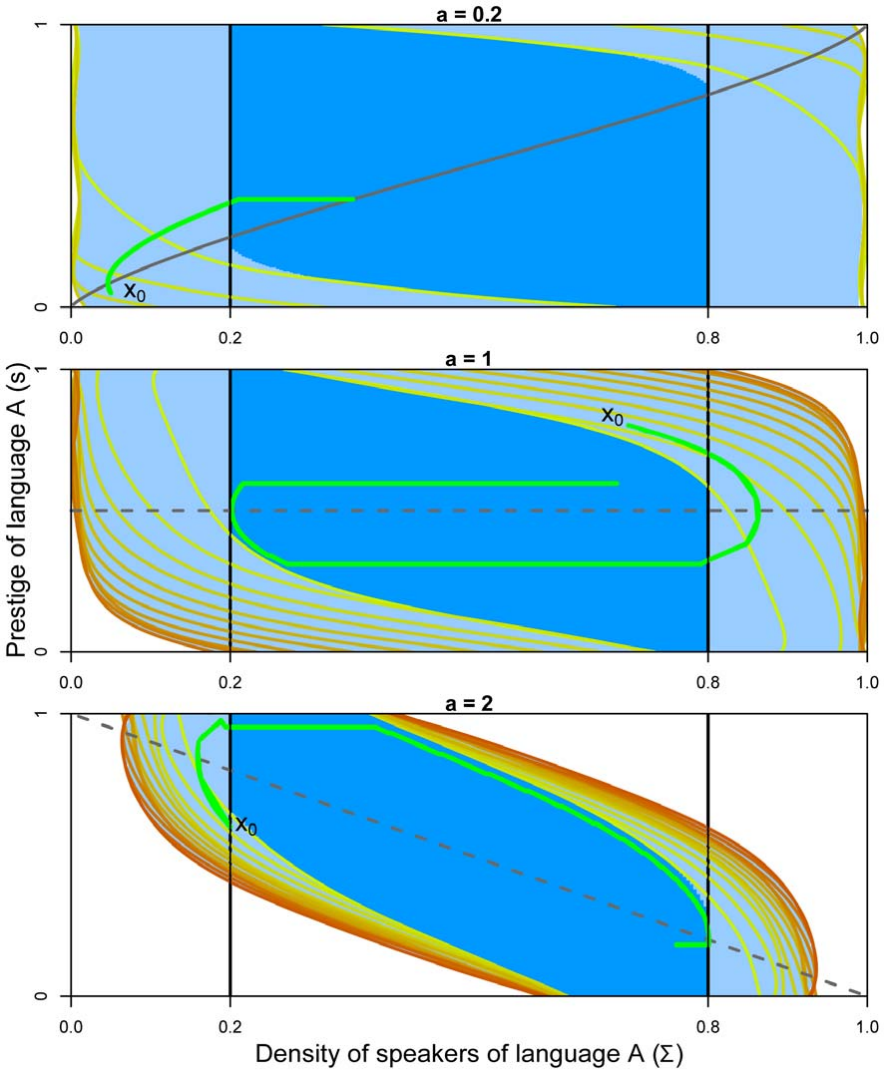
Examples of trajectories (in green) starting from a point 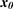 during 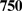 time steps, that allow the system to restore its viability at the minimal cost of restoration, using cost function Eqn 12. The continuous grey line represents stable fixed points and the dotted grey line unstable fixed points. Note that for an initial state 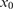 located inside 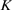 but outside 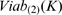, the trajectory crosses the viability constraint set boundaries before reaching 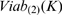.

Figure 7 displays some trajectories starting from resilient states for 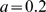, 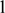 and 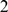. Considering the cost 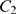 of Eqn 12, the controller produces a trajectory that avoids situations where the density of speakers is too small or too large, because these are the most costly. Notice that for 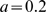, the trajectory first reaches the equilibrium line outside 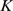, but in order to bring the system inside the viability kernel, the control function is chosen such that it does not get stuck on this fixed point. The procedure leads the system to a second fixed point, located this time inside the viability kernel. Even if the starting point is located inside 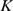 but outside the viability kernel (see for example case 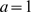), the trajectory crosses the viability constraint set before going back to 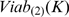, as it is not possible by definition for these states to directly reach the viability kernel.

### Conclusion

In this paper, we provide general means for determining action policies to maintain the coexistence of two languages in competition within the Abrams-Strogatz model [12] by using the framework of viability theory. We compute viable policies of action on the prestige variable to keep language coexistence within a given constraint set, computing the viability kernel of the system. We thus give evidence of the Abrams and Strogatz remark: language coexistence is unstable if we consider a fixed prestige, but introducing the prestige as a control variable of the model enables the maintenance of a bilingual society, where both languages have a density above a critical value. We also define the resilience of the system in the formalism of viability theory: the system is resilient to a perturbation if, after the perturbation, there exists an action policy driving back the system to its viability kernel. In this way, we determine the action policies that minimize the cost to drive an endangered language to coexistence (i.e. to the viability kernel of the system). In the paper, we have analyzed the role played by the two parameters of the model: the prestige of the language, 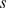, and the volatility, 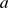. The prestige has been considered as the control variable of the system; we have shown how the viability kernel shrinks as the volatility parameter increases, due to the fact that agents become less likely to change their language.

The whole approach illustrates the new definition of resilience proposed in Ref [25], which enlarges previous definitions of resilience, yet with a precise mathematical meaning. In particular, we don't need to define the resilience relatively to the attractors of the dynamics, whereas the presence of such attractors is generally required in previous mathematical views of resilience [26], [27]. In the future, it will be interesting to consider the extension of the Abrams-Strogatz model that includes bilingual speakers [19], [23], and compare the results with the ones presented in this paper in order to illustrate which is the role of bilingual agents in the dynamics of language competition from the viability theory perspective.

## Supporting Information

Appendix S1 Theoretical bounds of the Abrams-Strogatz model (system Eqn 7) associated with the viability constraint set Eqn 8.
(0.06 MB PDF)

Appendix S2 Theoretical bounds of the Abrams-Strogatz model (system Eqn 9) associated with the viability constraint set Eqn 10.
(0.09 MB PDF)

## References

[1] Nettle D (1999). Using social impact theory to simulate language change.. Lingua.

[2] Nowak MA, Komarova NL, Niyogi P (2001). Evolution of universal grammar.. Science.

[3] Crystal D (2000). Language death.

[4] Weinreich U (1953). Languages in contact.

[5] Tsunoda T (2005). Language endangerment and language revitalisation.

[6] O'Driscoll J (2001). A face model of language change.. Multilingua.

[7] De Bot K, Stoessel S (2002). Introduction: language change and social networks.. International Journal of the Sociology of Language.

[8] Schulze C, Stauffer D (2005). Monte carlo simulation of the rise and the fall of languages.. International Journal of Modern Physics C.

[9] de Oliveira VM, Gomes MAF, Tsang IR (2006). Theoretical model for the evolution of the linguistic diversity.. Physica A.

[10] Schulze C, Stauffer D (2006). Recent developments in computer simulations of language competition.. Computing in Science and Engineering.

[11] Schulze C, Stauffer D, Wichmann S (2008). Birth, survival and death of languages by monte carlo simulation.. Communication in Computional Physics.

[12] Abrams D, Strogatz S (2003). Modelling the dynamics of language death.. Nature.

[13] Stauffer D, Castelló X, Eguíluz VM, San Miguel M (2007). Microscopic abrams-strogatz model of language competition.. Physica A.

[14] Patriarca M, Leppänen T (2004). Modeling language competition.. Physica A.

[15] Patriarca M, Heinsalu E (2008). Influence of geography on language competition.. Physica A.

[16] Pinasco J, Romanelli L (2005). Coexistence of languages is possible.. Physica A.

[17] Mira J, Paredes A (2005). Interlinguistic similarity and language death dynamics.. Europhysics Letters.

[18] Wang W, Minett J (2005). The invasion of language: emergence, change and death.. Trends in Ecology and Evolution.

[19] Castelló X, Eguíluz VM, San Miguel M (2006). Ordering dynamics with two non-excluding options: bilingualism in language competition.. New Journal of Physics.

[20] Castelló X, Toivonen R, Eguíluz VM, Saramäki J, Kaski K (2007). Anomalous lifetime distributions and topological traps in ordering dynamics.. Europhysics Letters.

[21] Toivonen R, Castelló X, Eguíluz VM, Saramäki J, Kaski K (2009). Broad lifetime distributions for ordering dynamics in complex networks.. Physical Review E.

[22] Labov W (1972). Sociolinguistic Patterns.

[23] Minett J, Wang W (2008). Modelling endangered languages: the effects of bilinguism and social structure.. Lingua.

[24] Aubin JP (1991). Viability theory.

[25] Martin S (2004). The cost of restoration as a way of defining resilience: a viability approach applied to a model of lake eutrophication.. Ecology and Society.

[26] Pimm S (1984). The complexity and stability of ecosystems.. Nature.

[27] Ludwig J, Walker B, Holling C (1997). Sustainability, stability and resilience.. Conservation Ecology.

[28] Holley R, Liggett T (1975). Ergodic theorems for weakly interacting systems and the voter model.. Annals of Probability.

[29] Suchecki K, Eguíluz VM, San Miguel M (2005). Voter model dynamics in complex networks: Role of dimensionality, disorder, and degree distribution.. Physical Review E.

[30] Castellano C, Vilone D, Vespignani A (2003). Incomplete ordering of the voter model on small-world networks.. Europhysics Letters.

[31] Vazquez F, Eguíluz VM (2008). Analytical solution of the voter model on uncorrelacted networks.. New Journal of Physics.

[32] Wurm SA (2001). Atlas of the world's languages in danger of disappearing..

[33] Chapel L, Martin S, Deffuant G (2007). Lake eutrophication: using resilience evaluation to compute sustainable policies..

[34] Deffuant G, Chapel L, Martin S (2007). Approximating viability kernels with support vector machines.. IEEE transactions on automatic control.

