# Supplementary material for: Viability and Resilience of Languages in Competition
Source: PLoS One. 2010 Jan 26;5(1):e8681. doi: 10.1371/journal.pone.0008681 (PMC2811194; doi:10.1371/journal.pone.0008681)
Supplement: Appendix S1 — Theoretical bounds of the Abrams-Strogatz model (system Eqn 7) associated with the viability constraint set Eqn 8. (0.06 MB PDF) [file pone.0008681.s001.pdf]

# Appendix S1. Theoretical bounds of the Abrams-Strogatz model (system Eqn 7) associated with the viability constraint set Eqn 8

## Case 1: $a = 1$

In this case, we have $Viab_{(1)}(K) = K$

*PROOF.*

Equation $\begin{cases} \frac{d\Sigma}{dt} = (1-\Sigma)\Sigma(\Sigma^{a-1}s - (1-\Sigma)^{a-1}(1-s)) \\ s = u \ ; \ u \in \{-c, +c\} \end{cases}$ can be rewritten as:

$$\frac{d\Sigma}{dt} = (2u-1)\Sigma(1-\Sigma). \tag{1}$$

For $0.2 \leq \Sigma \leq 0.8$, we have $\Sigma(1-\Sigma) > 0$. Thus, with $u = 0.4$, we have $\frac{d\Sigma}{dt} < 0$ and with $u = 0.6$, we have $\frac{d\Sigma}{dt} > 0$.
Then, for all the states $\Sigma \in K$, there exists at least one control function that maintains the system inside $K$, and all the states are viable. □

## Case 2: $a \neq 1$

For $\Sigma \in K$, $\frac{d\Sigma}{dt} = 0 \Leftrightarrow \Sigma = \left(\left(\frac{u}{1-u}\right)^{\frac{1}{a-1}} + 1\right)^{-1} = E_u$, with $u = s$.

### Case 2.1: $a < 1$

In this case, we have $Viab_{(1)}(K) = K$.

*PROOF.*

For $0.2 \leq \Sigma \leq 0.8$, $\forall u \in \{0.4, 0.6\}$, the equilibria are stable (see subsection Language Dynamics: the Abrams-Strogatz Model). In addition, it can be easily shown that, for $u \in \{0.4, 0.6\}$, $E_u \in K$. Thus, $\forall u$, the dynamics leads to a stable fixed point $E_u \in K$. □

### Case 2.2: $a > 1$

In this case, we have $Viab_{(1)}(K) = \{\Sigma \in K \text{such that } E_{0.6} \leq \Sigma \leq E_{0.4}\}$.

*PROOF.*

- For all the points located inside the viability kernel, there exists one control that allows the system to stay inside the viability kernel.
  For $\Sigma \in Viab_{(1)}(K)$, we have $\frac{d\Sigma}{dt} < 0$ for $u = 0.4$ and $\frac{d\Sigma}{dt} > 0$ for $u = 0.6$.
- For all the points located outside the viability kernel, there is no control that allows the system to return to the viability kernel.
  For $\Sigma < E_{0.6}$, we have $\frac{d\Sigma}{dt} < 0$ for $u = 0.4$ and $\frac{d\Sigma}{dt} < 0$ for $u = 0.6$ ($\Sigma \to 0$).
  For $\Sigma > E_{0.4}$, we have $\frac{d\Sigma}{dt} > 0$ for $u = 0.4$ and $\frac{d\Sigma}{dt} > 0$ for $u = 0.6$ ($\Sigma \to 1$).

□
