# Supplementary material for: Viability and Resilience of Languages in Competition
Source: PLoS One. 2010 Jan 26;5(1):e8681. doi: 10.1371/journal.pone.0008681 (PMC2811194; doi:10.1371/journal.pone.0008681)
Supplement: Appendix S2 — Theoretical bounds of the Abrams-Strogatz model (system Eqn 9) associated with the viability constraint set Eqn 10. (0.09 MB PDF) [file pone.0008681.s002.pdf]

Appendix S2. Theoretical bounds of the Abrams-Strogatz model (system Eqn 9) associated with the viability constraint set Eqn 10

We remind that the dynamics $\left(\frac{d\Sigma}{dt}, \frac{ds}{dt}\right) = F(\Sigma, s, u)$ are defined by:

$$\begin{cases} \dfrac{d\Sigma}{dt} = F(\Sigma, s, u) = (1-\Sigma)\Sigma\left(\Sigma^{a-1}s - (1-\Sigma)^{a-1}(1-s)\right) \\ \dfrac{ds}{dt} = u \\ u \in [-0.1, 0.1] \end{cases} \tag{1}$$

and that $K = [\underline{\Sigma}, \overline{\Sigma}] \times [0,1]$ is the viability constraint set.

We aim at finding explicit formulas for $Viab_F(K)$, the viability kernel under the dynamics F. We first instroduce two functions f_1 and f_2 and then prove that these functions enable us to define a set which is $Viab_F(K)$.

Definition of f_1 and f_2

- Let $C_1 = \{(\Sigma(t), s(t)), t \in [0; +\infty[\}$ satisfying

$$\begin{cases} \dfrac{d\Sigma(t)}{dt} = -(1-\Sigma(t))\Sigma(t)\left(\Sigma^{a-1}(t)s(t) - (1-\Sigma(t))^{a-1}(1-s(t))\right) \\ \dfrac{ds(t)}{dt} = 0.1 \\ \Sigma_1 = 0.8 \text{ and } s_1(0) = s_1 = \dfrac{0.2^{a-1}}{0.8^{a-1} + 0.2^{a-1}} \end{cases} \tag{2}$$

where $\Sigma(t)$ is the density of A-speakers at time t and $s(t)$ the prestige at time t.
We have $C_1 = \left\{(\Sigma, s) \in \mathbb{R}^2 | \Sigma = f_1(s), s \geq s_1\right\}$ with:

$$f_1(s) = \Sigma_1 + \int_{s_1}^{s} -(1-f_1(\widetilde{s}))f_1(\widetilde{s})(f_1(\widetilde{s})^{a-1}\widetilde{s} - (1-f_1(\widetilde{s}))^{a-1}(1-\widetilde{s}))d\widetilde{s}. \tag{3}$$

Note that $f_1'(s_1) = 0$ and that $f_1''(s) < 0$ when $f_1(s) \in [0.2, 0.8]$ and $f_1'(s) = 0$. Consequently, $f_1'(s) < 0$ when $s > s_1$ and $f_1(s) \in [0.2, 0.8]$.

- Let $C_2 = \{(\Sigma(t), s(t)), t \in [0; +\infty[\}$ satisfying:

$$\begin{cases} \dfrac{d\Sigma(t)}{dt} = -(1-\Sigma(t))\Sigma(t)\left(\Sigma^{a-1}(t)s(t) - (1-\Sigma(t))^{a-1}(1-s(t))\right) \\ \dfrac{ds(t)}{dt} = -0.1 \\ \Sigma_2 = 0.2 \text{ and } s_2(0) = s_2 = \dfrac{0.8^{a-1}}{0.2^{a-1} + 0.8^{a-1}} \end{cases} \tag{4}$$

We have $C_2 = \left\{(\Sigma, s) \in \mathbb{R}^2 | \Sigma = f_2(s), s \leq s_2\right\}$ with:

$$f_2(s) = \Sigma_2 - \frac{1}{0.1}\int_{s_2}^{s} -(1-f_2(\widetilde{s}))f_2(\widetilde{s})(f_2(\widetilde{s})^{a-1}\widetilde{s} - (1-f_2(\widetilde{s}))^{a-1}(1-\widetilde{s}))d\widetilde{s}. \tag{5}$$

Note that $f_2'(s_2) = 0$ and that $f_2''(s) > 0$ when $f_2(s) \in [0.2, 0.8]$ and $f_2'(s) = 0$. Consequently, $f_2'(s) < 0$ when $s < s_2$ and $f_2(s) \in [0.2, 0.8]$.

Definition of $Viab_F(K)$ and proofs

Theorem . *Let $E \subset K$ the subset defined by:*

$$\left\{ \quad (\Sigma, s) \in K \quad \middle| \quad \begin{array}{l} \Sigma \leq f_1(s) \text{ if } s \geq s_1(0) \\ \Sigma \geq f_2(s) \text{ if } s \leq s_2(0) \end{array} \quad \right\} \tag{6}$$

then we have $E = Viab_F(K)$.

PROOF PART 1: E is a viability domain: all the points inside E are viable.

We have to prove that for all $(\Sigma, s) \in \partial E$ (where ∂E is the boundary of the subset E), there exists at least one control u such that $F(\Sigma, s, u)$ belongs to the tangent cone of E at the point (Σ, s), denoted $T_E(\Sigma, s)$.

Let $(\Sigma, s) \in \partial E$,

- if $\Sigma = 0.2$, as $f_2'(s) < 0$ when $s < s_2$ and $f_2(s) \in [0.2, 0.8]$, necessarily $s \geq s_2$. Moreover, $s \leq \min(1, f_1^{-1}(0.2))$. If $s = s_2$, $F(\Sigma, s, 0) = 0 \in T_E(\Sigma, s)$, if $s_2 < s < \min(1, f_1^{-1}(0.2))$, $F(\Sigma, s, u) \in T_E(\Sigma, s)$ for all $u \in [-0.1, 0.1]$.
- if $s = 1$, or if $(\Sigma, s) \in C_1$, $\Sigma < 0.8$, $F(\Sigma, s, -0.1) \in T_E(\Sigma, s)$.
- if $\Sigma = 0.8$, as $f_1'(s) < 0$ when $s > s_1$ and $f_1(s) \in [0.2, 0.8]$, necessarily $s \leq s_1$. Moreover, $s \geq \max(1, f_2^{-1}(0.8))$. If $s = s_1$, $F(\Sigma, s, 0) = 0 \in T_E(\Sigma, s)$, if $\max(1, f_2^{-1}(0.8)) < s < s_1$, $F(\Sigma, s, u) \in T_E(\Sigma, s)$ for all $u \in [-0.1, 0.1]$.
- if $s = 0$, or if $(\Sigma, s) \in C_2$, $\Sigma > 0.2$, $F(\Sigma, s, +0.1) \in T_E(\Sigma, s)$.

$\square$

PROOF PART 2: E is the largest viability domain.

Let's first introduce some notations:

- Let $(\overline{\Sigma}, \overline{s}) \in K \backslash E$. We can suppose $\overline{s} > f_1^{-1}(\overline{\Sigma})$. The argument is the same if $\overline{s} > f_2^{-1}(\overline{\Sigma})$.
- Let $(\overline{\Sigma}(t), \overline{s}(t)), t \in [0; +\infty[$ an evolution starting from $(\overline{\Sigma}, \overline{s})$ and satisfying Eqn 1.
- Let $(\Sigma^*(t), s^*(t)), t \in [0; +\infty[$ defined by:

$$\left\{ \begin{array}{l} \dfrac{d\Sigma^*(t)}{dt} = (1 - \Sigma^*(T))\Sigma^*(T)\left(\Sigma^{*a-1}(T)s^*(T) - (1 - \Sigma^*(t))^{a-1}(1 - s^*(t))\right) \\ \dfrac{ds^*(t)}{dt} = -0.1 \\ \Sigma^*(0) = \overline{\Sigma} \text{ and } s^*(0) = f_1^{-1}(\overline{\Sigma}) \end{array} \right. \tag{7}$$

Then, $(\Sigma^*(0), s^*(0)) \in C_1$ and there exists T such that $(\Sigma^*(T), s^*(T)) = (\Sigma_1, s_1)$ and $(\Sigma^*(t), s^*(t)) \in C_1, \forall t \in [0; T]$.
We have $\overline{s}(0) > s^*(0)$ and as $s^{*'}(t) = -0.1$ and $\overline{s}'(t) = u \in [-0.1, 0.1]$, $\forall t \in [0; T], \overline{s}(t) > s^*(t)$. Furthermore, $\overline{\Sigma}(0) = \Sigma^*(0)$ and $\frac{d\overline{\Sigma}}{dt}(0) = F(\overline{\Sigma}(0), \overline{s}(0)) > F(\Sigma^*(0), s^*(0)) = \frac{d\Sigma^*}{dt}(0)$ so there exists $\widehat{t} > 0$ such that $\overline{\Sigma}_A(t) > \Sigma_A^*(t)$ for all $t \in]0, \widehat{t}]$.

Assume that there exists $\widetilde{t} \in]\widehat{t}, T]$ such that $\overline{\Sigma}_A(t) > \Sigma_A^*(t)$ for all $t \in]\widehat{t}, \widetilde{t}]$ and $\overline{\Sigma}_A(\widetilde{t}) > \Sigma_A^*(\widetilde{t})$. Then $\frac{d\overline{\Sigma}_A}{d}(\widetilde{t}) \leq \frac{d\Sigma^*}{d}(\widetilde{t})$ but $\frac{d\overline{\Sigma}_A}{d}(\widetilde{t}) = F(\overline{\Sigma}_A(\widetilde{t}), \overline{s}(\widetilde{t})) > F(\Sigma_A^*(\widetilde{t}), s^*(\widetilde{t})) = \frac{d\Sigma^*}{d}(\widetilde{t})$ since $\overline{\Sigma}_A(\widetilde{t}) = \Sigma_A^*(\widetilde{t})$ and $\overline{s}(\widetilde{t}) > s^*(\widetilde{t})$. Hence the contradiction, so $\forall t \in [0, T]$, $\overline{\Sigma}_A(t) > \Sigma^*(t)$.

Consequently, $(\overline{\Sigma}_A(T), \overline{s}(T)) \notin K$ and $(\overline{\Sigma}_A(T), \overline{s}(T)) \notin Viab_F(K)$. $\square$
